# Hippocampal Metastasis Rate Based on Non-Small Lung Cancer TNM Stage and Molecular Markers

**DOI:** 10.3389/fonc.2022.781818

**Published:** 2022-05-10

**Authors:** Sung Jun Ahn, Hyeokjin Kwon, Jun Won Kim, Goeun Park, Mina Park, Bio Joo, Sang Hyun Suh, Yoon Soo Chang, Jong-Min Lee

**Affiliations:** ^1^Department of Radiology, Gangnam Severance Hospital, Yonsei University, College of Medicine, Seoul, South Korea; ^2^Department of Electronic Engineering, Hanyang University, Seoul, South Korea; ^3^Department of Radiation Oncology, Gangnam Severance Hospital, Yonsei University, College of Medicine, Seoul, South Korea; ^4^Biostatistics Collaboration Unit, Yonsei University College of Medicine, Seoul, South Korea; ^5^Department of Internal Medicine, Gangnam Severance Hospital, Yonsei University, College of Medicine, Seoul, South Korea; ^6^Department of Biomedical Engineering, Hanyang University, Seoul, South Korea

**Keywords:** Non-small cell lung cancer, Brain metastasis, Hippocampal-avoidance whole-brain radiation therapy, Epidermal growth factor receptor, lung-cancer stage

## Abstract

Hippocampal-avoidance whole-brain radiation therapy (HA-WBRT) is justified because of low hippocampal brain metastases (BM) rate and its prevention of cognitive decline. However, we hypothesize that the risk of developing BM in the hippocampal-avoidance region (HAR) may differ depending on the lung-cancer stage and molecular status. We retrospectively reviewed 123 patients with non-small cell lung cancer (NSCLC) at the initial diagnosis of BM. The number of BMs within the HAR (5 mm expansion) was counted. The cohort was divided into patients with and without BMs in the HAR, and their clinical variables, TNM stage, and epidermal growth factor receptor (EGFR) status were compared. The most influential variable predicting BMs in the HAR was determined using multi-variable logistic regression, classification and regression tree (CART) analyses, and gradient boosting method (GBM). The feasibility of HAR expansion was tested using generalized estimating equation marginal model. Patients with BMs in the HAR were more frequently non-smokers, and more likely to have extra-cranial metastases and EGFR mutations (p<0.05). Multi-variable analysis revealed that extra-cranial metastases were independently associated with the presence of BM in the HAR (odds ratio=8.75, p=0.04). CART analysis and GBM revealed that the existence of extra-cranial metastasis was the most influential variable predicting BM occurrence in the HAR (variable importance: 23% and relative influence: 37.38). The estmated BM incidence of patients without extra-cranial metastases in th extended HAR (7.5-mm and 10-mm expansion) did not differ significantly from that in the conventional HAR. In conclusion, NSCLC patients with extra-cranial metastases were more likely to have BMs in the HAR than those without extra-cranial metastases.

## Introduction

The brain is a common site of distant metastasis in patients with non-small cell lung cancer (NSCLC) (1, 2). The overall incidence of brain metastases (BMs) is estimated to be 10-20% at initial presentation, but can increase up to 40% throughout the clinical course (3, 4). Recently, epidermal growth factor receptor (EGFR) gene mutations and anaplastic lymphoma kinase (ALK) rearrangements have been identified as significant risk factors for the development of BMs (5–7). Although molecular targeted therapy against mutated driver oncogene such as EGFR or ALK has improved the survival rates of patients with lung cancer, BMs remain an important cause of morbidity and are associated with progressive neurologic deficits (8, 9).

Whole-brain radiation therapy (WBRT) has been the standard of care for patients with multiple BMs. However, most patients experience cognitive deterioration after WBRT, which raises concerns about its toxicity (10–12). Accumulating evidence suggests that neural stem cells in the hippocampus are exquisitely sensitive to therapeutic doses of cranial radiation, which is believed to be a key mechanism underlying the cognitive decline after WBRT (13–15). Intensity-modulated radiotherapy techniques, developed to avoid the hippocampal neural stem-cell niche during WBRT to prevent these adverse effects, reduce the mean dose to this neural stem-cell compartment by ≥80% (15, 16). An international multi-institutional single-arm phase II trial (RTOG 0933) showed that hippocampal-avoidance WBRT (HA-WBRT) prevented cognitive decline in patients with BMs (17). In this study, the mean relative decline in Hopkins Verbal Learning Test-Revised Delayed Recall at 4 months was 7.0%, significantly lower in comparison with that of historical controls treated with WBRT (30%). A prospective multi-institutional randomized phase III trial (NRG CC001) demonstrated that HA-WBRT plus memantine prevented deterioration in executive function at 4 months (23.3% vs. 40.4%) and learning and memory function at 6 months (11.5% vs. 24.7%), as well as alleviated patient-reported symptoms, with no difference in the intracranial progression-free survival and overall survival compared with those treated with WBRT plus memantine (18). On the basis of a recent phase III study of 150 patients with small cell lung cancer, the HA-PCI arm showed a lower decline in the delayed free recall test after 3 months compared with the standard PCI arm (5.8% vs. 23.5%). However, incidence of BMs, overall survival and quality of life were not significantly different between groups (19). Few proactive studies have indicated extending the radiation protected zone from the hippocampus to the limbic system, which is known to regulate memory and emotions (20, 21).

However, for patients with multiple BMs, HA-WBRT is accompanied by the possibility of BMs occurring in the hippocampus after HA-WBRT, leading to treatment failure. Few studies have estimated the risk of metastases in the hippocampal-avoidance region (HAR). A pioneer study with 371 BMs from several primary cancers supported the use of HA-WBRT owing to a low BM rate in the HAR (8.6% of patients) (22). However, the cohort of this study was limited to patients with ≤10 BMs where stereotactic radiosurgery might be preferred over WBRT. A more recent study with 116 patients with BMs reported a slightly higher risk of BMs (11.2%) in the HAR (23). However, it did not conduct risk stratification for the development of BMs in this region. Information about the incidence of hippocampal BM according to the lung cancer stage or EGFR/ALK mutation status can serve as a good guide for the indication for HA-WBRT. Thus, this study aimed to estimate the incidence of BM in the hippocampus in patients with NSCLC as stratified according to the 8th edition of the American Joint Committee on Cancer (AJCC) staging system and EGFR/ALK mutation status.

## Materials and Methods

### Participants

This retrospective study was approved by our institutional review board, which waived the requirement for informed patient consent. We retrospectively searched the electronic medical records to identify lung cancer patients undergoing brain magnetic resonance imaging (MRI) for evaluation of BMs between April 2017 and October 2020. From 1092 available brain MRIs, we excluded 969 for the following reasons: (1) negative BMs (n=633), (2) history of neurosurgery or brain radiation therapy (n=283), (3) presence of other malignant diseases (n=24), (4) primary small cell lung cancer (n=22) and (5) motion or dental material artifacts on the MRIs (n=7). Finally, 123 NSCLC patients with 123 brain MR images showing BMs were included in this study. BMs were considered to be positive when (1) the clinical-radiologic consensus was compatible with BMs, (2) BM was confirmed at pathology with stereotactic biopsy or metastasectomy, (3) lesions suspected to be BMs increased in size at follow-up MRI or (4) decrease in size with treatment (4). The histopathological diagnoses of lung cancer were obtained using bronchoscopic, percutaneous needle-guided, or surgical biopsies for all patients. To determine the EGFR mutation status, DNA was extracted using a DNeasy isolation kit (Qiagen, Valencia, CA, USA) from FFPE tissues according to the manufacturer’s instructions. For the EGFR gene, direct DNA sequencing of exons 18 through 21 or PNAClampTM EGFR Mutation Detection Kit (PANAGENE, Daejeon, Korea) was performed. Each case was classified as positive or negative for a mutation based on comparison with the wild-type sequence. To identify ALK and ROS1 rearrangements, fluorescent *in situ* hybridization was performed using a break-apart ALK or ROS1 probe (Vysis LSI Dual Color, Break Apart Rearrangement Probe; Abbott Molecular, Abbot Park, IL, USA), respectively. ALK or ROS1 rearrangements were scored as positive when more than 15% of tumor cells displayed split signals or isolated signals containing a kinase domain (red for ALK and green for ROS1).

All data were completely anonymized, and all experiments were conducted in accordance with the approved guidelines.

### Staging

NSCLC staging was performed according to the 8th edition of AJCC guidelines without considering the BMs (24). The tumor–node–metastasis (TNM) stage at the diagnosis of lung cancer was based on computed tomography (CT) scans of the chest and abdomen, whole-bone scanning, and positron-emission tomography-CT, which were acquired as a part of initial evaluation for NSCLC. Extra-cranial metastasis at BM occurrence was based on the last CT and PET-CT work-up before the detection of BMs. Extra-cranial metastasis was defined as (1) tumor in the contralateral lung, (2) pleural/pericardial nodule, malignant effusion, or (3) extra-thoracic metastasis other than BMs.

### MRI and Preprocessing

Routine MRIs for the evaluation of BMs were acquired using the Siemens 3T Vida (Siemens Healthineers, Erlangen, Germany) or GE 3T Discovery MR750 (GE Healthcare, Milwaukee, WI, USA) scanner. Our brain MRI protocol for the Siemens 3T scanner included the acquisition of T1-weighted three-dimensional (3D) magnetization-prepared rapid acquisition with gradient echo (MPRAGE) imaging. A 3D turbo spin-echo T1-weighted image (SPACE) was acquired after administering gadobutrol 0.2 mmol/kg (Gadovist, Bayer Schering Pharma; Berlin, Germany). The sequence parameters for the 3D T1-weighted MPRAGE were as follows: inversion time (TI)=900 ms, echo time (TE) 3 ms, repetition time (TR)=2300 ms, flip angle=9°, slice thickness=1 mm, field-of-view (FOV)=256 mm, matrix=256×256, slice thickness=1 mm, generalized autocalibrating partial parallel acquisition=2, and time of acquisition=5 min 12 s. The sequence parameters for 3D T1-weighted SPACE were as follows: TE=33 ms, TR=700 ms, slice thickness=0.8 mm, FOV=230 mm, matrix=288×288, slice thickness=0.8 mm, acceleration factor of compressed sensing=9, and time of acquisition=3 min 44 s. A corresponding sequence was used with similar MR parameters for the GE scanner.

MRIs were processed using the FMRIB Software Library (http://www.fmrib.ox.ac.uk/fsl). A neuroradiologist labeled the BMs by manually segmenting the 3D BM volumes on the native 3D T1-weighted SPACE images. The binary labels of the BMs were transformed into the Montreal Neurological Institute (MNI) space by co-registering the 3D T1-weighted MPRAGE images to the gadolinium-enhanced 3D T1-weighted SPACE images using rigid body transformation. The native 3D T1-weighted MPRAGE images were converted to the standard MNI 152 T1-1-mm brain model using an affine transform matrix. The estimated transform matrix was concatenated with a co-registration transform matrix, and the resultant matrix was applied to 3D T1-weighted SPACE images and binary labeled BM images. The voxel for the center of gravity was localized for each BM, and binary BM masks were regenerated as spheres with a 5-mm radius to consider the origin of the BMs more accurately and to standardize their chronological development (25).

**Figure 1:**
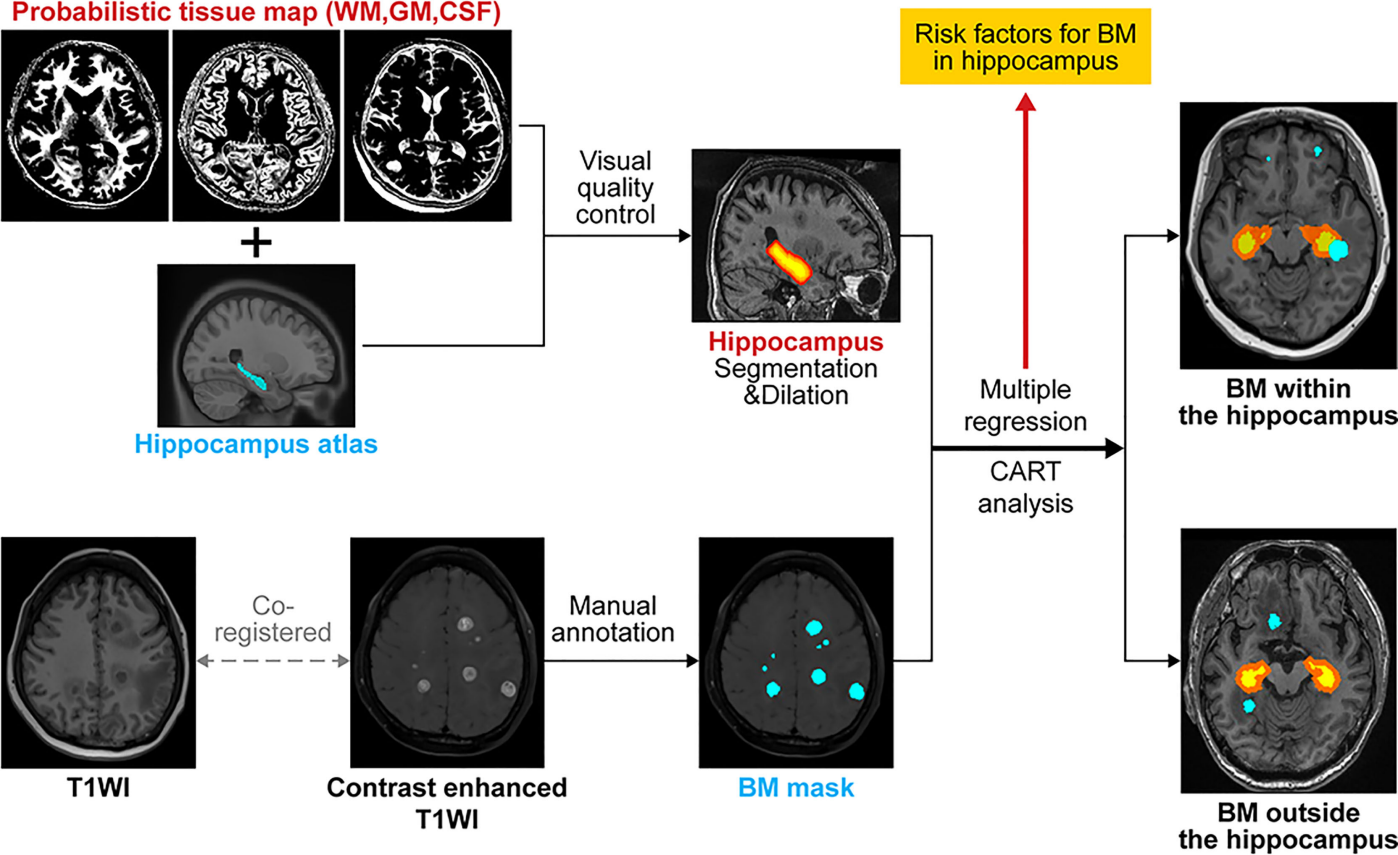
Schematic illustration of the flow of image analysis. BM, brain metastasis; WM, white matter; GM, gray matter; CSF, cerebrospinal fluid; CART, classification and regression tree analysis; T1WI, T1 weighted imaging.

An atlas-based graph cuts algorithm was utilized to define the hippocampal region (24). First, a hippocampal atlas was manually drawn on the International Consortium for Brain Mapping 152 template. An artificial neural network classifier was used to classify the gray matter, white matter, and cerebrospinal fluid regions (26). Subsequently, voxel-wise partial volume effects (PVEs) were estimated using the trimmed minimum covariance determinant method and the PVE maps were used as a prior information for the subsequent graph cuts segmentation (27). A hippocampal foreground mask was obtained using the graph cuts method, followed by the implementation of morphological opening to limit the false positives. We empirically applied the 25% threshold level to binarize the above-mentioned atlases by visual inspection for quality checks. The HAR was generated by volumetrically expanding the outline of hippocampal mask by 5 mm to account for systematic setup errors and dose falloff between the clinical target volume for the whole brain and hippocampus (15). Consequently, we generated two additional hippocampal masks by dilating the original hippocampal mask with 7.5-mm and 10-mm margins to investigate the possibility of expanding the HAR. Lastly, we counted the number of BM samples in each hippocampal mask (**Figure 1**).

### Statistical Analyses

The study cohort was divided into two groups: those with BMs in the HAR and those without BMs in the HAR. History of smoking, age, sex, T stage, N stage, M stage, TNM stage, extra-cranial metastasis at BM occurrence, histology, and EGFR/ALK/ROS1 mutation status were compared between the two groups. Independent t-tests were used for continuous variables, while the chi-squared test or Fisher’s exact test was used for categorical variables. Multi-variable logistic regression analysis with backward selection was also performed to adjust for the smoking history, extra-cranial metastasis at BM occurrence, and EGFR mutation status, which were statistically significant in the univariate analysis for the comparison between the presence and absence of BM in the HAR. A decision tree model distinguishing the presence of BM in the HAR from the absence of BM in the HAR was built using classification and regression tree (CART) analysis. CART analysis selects the best predictor variable for splitting the data into two child nodes with maximal purity. The process is repeated recursively for each child node, until either the minimum size of the terminal node is reached, or no further split improves the purity of the terminal node (28). To provide a more accurate estimate of the responsible variable for BM in the HAR, a popular ensemble learning method, gradient boosting method (GBM) was additionally performed. GBM builds an ensemble of shallow and weak successive trees with each tree learning and improving on the previous (29). The proportion of BM between three HARs (hippocampus plus 5-mm margin, 7.5-mm margin, and 10-mm margin) in patients without extra-cranial metastases were compared using a generalized estimating equation (GEE) marginal model to test the feasibility of HAR expansion (30).

## Results

### Patient Characteristics

A total of 123 patients with NSCLC with BMs were included in this study. Demographics and clinical characteristics are summarized in **Table 1**. Patients with BMs were more frequently non-smokers (83.33% versus 54.29%, p=0.02), more likely to have extra-cranial metastases (94.44% vs. 66.67%, p=0.03), and more likely to have EGFR mutations (66.67% versus 41.11%, p=0.04) than those without BMs in the HAR. No significant differences in the age, sex, T stage, N stage, M stage, TNM stage, histology, ALK, and ROS1 status were observed between groups.

**Table 1:** Baseline characteristics and staging of patients with BMs from NSCLC.

		Hippocampal avoidance region		
	Total (N=123)	Absence of BM (N=105)	Presence of BM (N=18)	p-value
Smoking history				0.02*
Nonsmoker	72 (58.54)	57 (54.29)	15 (83.33)	
Smoker	51 (41.46)	48 (45.71)	3 (16.67)	
Age (yr)	66.66 ± 10.17	67.11 ± 10.28	64.00 ± 9.27	0.23
Sex				0.12
Male	81 (65.85)	72 (68.57)	9 (50.00)	
Female	42 (34.15)	33 (31.43)	9 (50.00)	
Detection of BM				0.07
At diagnosis of lung cancer	66 (53.66)	60 (57.14)	6 (33.33)	
During the course of disease	57 (46.34)	45 (42.86)	12 (66.67)	
Chemotherapy				0.21
No	87 (70.73)	77 (73.33)	10 (55.56)	
Yes	36 (29.27)	28 (26.67)	8 (44.44)	
T stage				0.61
T1a	1 (0.81)	1 (0.95)	0 (0.00)	
T1b	5 (4.07)	4 (3.81)	1 (5.56)	
T1c	9 (7.32)	7 (6.67)	2 (11.11)	
T2a	20 (16.26)	19 (18.10)	1 (5.56)	
T2b	13 (10.57)	11 (10.48)	2 (11.11)	
T3	20 (16.26)	18 (17.14)	2 (11.11)	
T4	55 (44.72)	45 (42.86)	10 (55.56)	
N stage				0.42
N0	25 (20.33)	24 (22.86)	1 (5.56)	
N1	6 (4.87)	5 (4.76)	1 (5.56)	
N2	38 (30.89)	32 (30.48)	6 (33.33)	
N3	54 (43.90)	44 (41.91)	10 (55.56)	
M stage				0.06
M0	46 (37.39)	43 (40.95)	3 (16.66)	
M1a	8 (6.50)	8 (7.62)	0 (0.0)	
M1b	7 (5.69)	5 (4.76)	2 (11.11)	
M1c	62 (50.41)	49 (46.67)	13 (72.22)	
TNM				0.41
Stage 1	9 (7.32)	8 (7.62)	1 (5.55)	
Stage 2	7 (5.69)	7 (6.67)	0 (0.00)	
Stage 3	29 (23.58)	27 (25.71)	2 (11.11)	
Stage 4	78 (63.41)	63 (60.00)	15 (83.34)	
Extra-cranial metastasis at BM occurrence				0.03*
Absence	36 (29.26)	35 (33.33)	1 (5.56)	
Presence	87 (70.74)	70 (66.67)	17 (94.44)	
Histology				0.22
Adenocarcinoma	98 (79.67)	81 (77.14)	17 (94.44)	
Squamous cell carcinoma	14 (11.38)	14 (13.33)	0 (0.00)	
Large cell carcinoma	11 (8.94)	10 (9.52)	1 (5.56)	
EGFR				0.04*
Wild type	59 (54.63)	53 (58.89)	6 (33.33)	
Mutation	49 (45.37)	37 (41.11)	12 (66.67)	
ALK				0.53
Negative	75 (92.59)	64 (94.12)	11 (84.62)	
Positive	6 (7.41)	4 (5.88)	2 (15.38)	
ROS1				
Negative	52 (100)	9 (100)	61 (100)	
Positive	0 (0.00)	0 (0.00)	0 (0.00)	

NSCLC, non-small cell lung cancer; EGFR, epidermal growth factor receptor; ALK, anaplastic lymphoma kinase; MRI, magnetic resonance imaging; BM, brain metastasis. Asterisk (*) indicates a p-value < 0.05.

### Most Influential Variable Predicting BM Occurrence in the HAR

**Figure 2:**
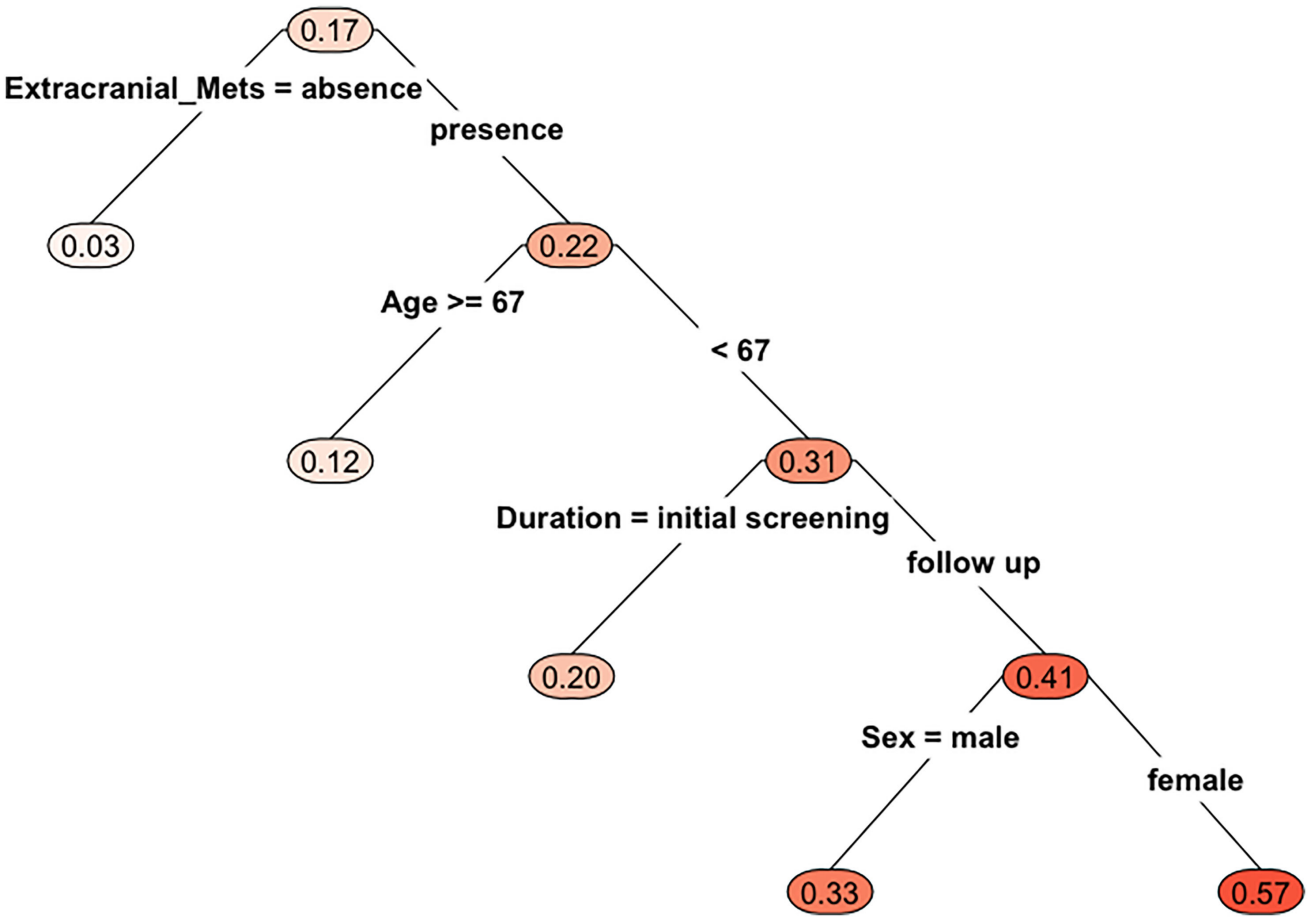
Results of the classification and regression tree analysis (CART). Mets, metastasis.

Multivariable analysis did not find any independent predictor for the presence of BM in the HAR (**Table 2**). However, the model using multivariable analysis with backward elimination found that extra-cranial metastasis at BM occurrence was independently associated with the presence of BM in the HAR [odds ratio (OR)=8.75; 95% confidence interval (CI): 1.64, 162.33, p=0.04, **Supplemental Table 1**]. Fifteen patients whose EGFR mutation status was unknown were excluded from the CART analysis. BMs were present in the HAR of 17% (18/108) of patients with NSCLC. The existence of extra-cranial metastasis was the first partitioning predictor in the decision tree model (**Figure 2**). BMs were found in the HAR in 22% (17/76) of patients of NSCLC with extra-cranial metastases, while 3% (1/32) of patients with NSCLC without extra-cranial metastases showed BMs in the HAR. Further branching was based on age of 67 years, followed by duration and sex. BMs were located in the HAR of 31% of patients with NSCLC with extra-cranial metastases and those aged <67 years (13/42), while 12% of patients with NSCLC with extra-cranial metastases and those aged >67 years (4/34) presented with BMs. BMs were observed in the HAR in 41% (9/22) of patients with NSCLC with extra-cranial metastases, age <67 years, and BMs detected during follow-up, while 20% of patients with NSCLC with extra-cranial metastases, age <67 years, and BMs detected at initial screening (4/20) presented with BMs. A total of 57% of female patients with NSCLC with extra-cranial metastases, age <67 years, and BMs detected during follow-up (3/7) demonstrated BMs in the HAR, while 33% of male patients with NSCLC with extra-cranial metastases, age <67 years, and BMs detected during follow-up (5/15) had BMs. The existence of extra-cranial metastasis at BM occurrence showed the highest variable importance score, followed by TNM stage, age, BMs during the course of disease, smoking history, histology, and EGFR mutation (**Table 3**). In addition, in GBM, existence of extra-cranial metastasis demonstrated the highest prediction power for BM occurrence in the HAR, followed by sex, BMs during the course of disease, age, TNM stage, EGFR mutation, smoking history and histology.

**Table 2:** Multiple logistic regression analysis for BM occurrence in the hippocampus plus 5-mm margin region.

	OR (95% CI)	p-value
Smoker	0.34 (0.07-1.31)	0.14
Extra-cranial metastasis at BM occurrence	7.82 (1.44-145.96)	0.05
EGFR mutation	1.68 (0.51-5.84)	0.39

EGFR, epidermal growth factor receptor; BM, brain metastasis.

**Table 3:** Variables for the prediction of BM incidence in the hippocampal avoidance region using classification and regression tree analysis and gradient boosting.

Cart analysis		Gradient boosting	
Variables	Importance(%)	Variables	Relative influence
Extra-cranial metastasis	25	Extra-cranial metastasis	37.38
TNM stage	22	Sex	31.84
Age	20	BMs during course of disease	14.96
BMs during course of disease	15	Age	12.91
Sex	11	TNM stage	2.39
Smoking history	5	EGFR mutation	0.34
Histology	1	Smoking history	0.14
EGFR mutation	1	Histology	0

BM, brain metastases.

### Feasibility of Expanding the Hippocampal Avoidance Region

We analyzed whether the number of BMs in the HAR increased when the margin of region was expanded from 5 to 7.5 or 10 mm using a GEE marginal model. This analysis was performed in 36 patients with NSCLC without extra-cranial metastases because this subgroup may be suitable for HA-WBRT. The estimated proportion of the incidence of BM in the HAR was 2.78% with a standard error of 2.75%, which did not differ significantly from that of the expanded regions (hippocampus plus 7.5 mm and hippocampus plus 10 mm, p>0.99, **Supplementary Table 2**).

## Discussion

This study tested the hypothesis that the hippocampal metastases rate would differ based on the patient characteristics, clinical stage, and molecular markers. Our results indicated that patients with BMs in the HAR were more likely to have extra-cranial metastases before BM occurrence. The clinical implication of this observation is important because of the possibility that BMs may occur in the hippocampus after the implementation of HA-WBRT in patients with extra-cranial metastases, leading to treatment failure. Thus, different radiation therapy strategies may be necessary depending on the existence of extra-cranial metastasis in patients with NSCLC. Moreover, our results demonstrated that the hippocampal metastasis rate was low in patients with NSCLC without extra-cranial metastasis and this rate remained low if the avoidance zone was expanded to the hippocampus plus 10-mm region. This observation is also clinically relevant because the application of HA-WBRT to a wider safe zone in patients without extra-cranial metastases may be instrumental in ensuring successful treatment while preventing cognitive decline.

A multi-institutional study with 371 patients with 1133 BMs found BMs within the HAR in 8.6% patients, showing that HA-WBRT may be suitable for controlling BMs (22). However, a more recent single-center analysis demonstrated a relatively high incidence of BMs within the HAR in patients with NSCLC (11.8%, 7/59) (23). In our study, 14% of patients with NSCLC (18/123) had BMs within the HAR. This higher incidence of BMs within the HAR in recent studies can be explained by the prolonged survival of patients with widespread intracranial disease as well as screening with improved MRI techniques, which enhanced the detection of BMs (31). Our results also demonstrated that 83.3% of patients with BMs in HAR (15/18) have extensive BMs (≥10). However, patient stratification by the existence of extra-cranial metastasis revealed significant differences in hippocampal metastasis: 19.5% of patients with extra-cranial metastases (17/87) had BMs in the HAR, while 2.7% of patients without extra-cranial metastases (1/36) had BMs in the HAR. Our multivariate analysis indicated that patients with extra-cranial metastasis are 8.75 times more likely to have BMs in the HAR. CART analysis and GBM also suggested that extra-cranial metastasis was the most influential variable predicting BM occurrence in the HAR. The mechanism underlying this phenomenon is unknown. However, the presence of extra-cranial metastasis has been known as an independent prognostic factor for the survival of patients with BMs (32, 33). In addition, BMs are commonly diagnosed as associated with extra-cranial metastases (34, 35). We may infer that this extra-cranial metastatic burden may increase the risk of BM occurrence in the HAR. Hence, we concluded that HA-WBRT application is safe in patients without extra-cranial metastases; however, caution must be exercised while applying HA-WBRT to patients with extra-cranial metastases; alternatively, a different treatment strategy must be adopted. Recently, stereotactic radiosurgery (SRS) has been preferred over WBRT for limited BMs because its efficacy is non-inferior with greater preservation of neurocognitive functioning, now being applied up to 10 BMs (36, 37). Few trials have investigated the treatment of > 10 BMs with SRS alone (38, 39). Based on our results, whether presence of extra-cranial metastases may increase the risk of treatment failure or not should be considered in these trials.

According to our CART analysis, apart from extra-cranial metastases, age, BMs during course of disease, and sex were important risk factors for BM occurrence in the hippocampus. Our study demonstrated that younger patients (age <67 years) had a higher hippocampal metastasis rate than that of their older counterparts (age ≥67 years). Generally, age is a risk factor for the development BM in NSCLC. Age <60-70 years was associated with the risk of BMs (40–43). We may assume that the cerebrovascular environment in younger patients differ from that in older patients, in addition to better outcomes with a longer survival in the former, leading to higher BM prevalence. Based on the same rationale, younger patients are likely to develop BMs in the hippocampus. The reason for the greater predominance of hippocampal BMs in women during the course of the disease than that at the initial diagnosis of lung cancer is unclear. Further studies are necessary to validate this observation.

Numerous studies have reported that patients with EGFR mutations have a nearly two-fold higher risk of BMs than those without EGFR mutation (44–47). Our univariate analysis revealed that the proportion of EGFR mutation was higher in patients with BMs in the HAR (12/18, 66.67%) than in patients without BMs in the HAR (37/105, 35.24%). The mechanism underlying this phenomenon may be associated with epithelial-mesenchymal transition, which may result in the increased motility and invasiveness of tumor cells (48, 49). However, according to our multivariate analysis, EGFR mutations were not an independent risk factor for BM occurrence in the HAR. ALK-rearrangement tumors exhibit aggressive behavior, including extra-thoracic metastases (50, 51), and have a higher risk of BMs. The cumulative incidence of BMs after diagnosis reaches 58% at 3 years (52, 53). Compared with ALK rearrangement, ROS1 rearrangement is associated with lower rates of extra-thoracic metastases and fewer BMs, but it may still increase the likelihood of BMs (54, 55). Our results demonstrated a higher but not significant proportion of ALK rearrangement (2/13, 15.38%) in patients with BM in the HAR than in patients without (4/68, 5.88%), and no significant difference in the incidence of ROS1 rearrangement between groups (0/61, 0% vs. 0/9, 0%). However, these results should be carefully interpreted because of fairly low incidence in our study.

Several studies have reported the incidence of progressive leukoencephalopathy following cranial radiation (56–58). WBRT injures the small cerebral vasculature and neuropil, resulting in oligodendrocyte death and demyelination (59, 60). Accumulating evidence suggests that diseases of the white matter are associated with neurocognitive dysfunction (61, 62). Injury to the parahippocampal white matter may contribute to memory decline as much as injury to the hippocampus itself (63). Thus, protecting the hippocampus as well as parahippocampal white matter from the radiation dose may prevent the development of neurocognitive dysfunction. Given the lower hippocampal metastasis rate in patients without extra-cranial metastasis, we investigated whether extending the radiation-safe zone was a safe practice. Our results demonstrated that the occurrence of BM in the hippocampus plus 10-mm region did not differ significantly from that in the hippocampus plus 5-mm region. Our results may be applied in planning radiation strategies with a greater safety margin.

Our study had a few limitations. First, the hippocampal metastases rate in our sample was measured before radiation therapy to BMs because the number of available patients who underwent follow-up MRI after HA-WBRT was small (n=26, **Supplemental Table 3**). Other patients were lost to follow-up after being diagnosed with BMs, were treated with SRS, or underwent surgery or conventional WBRT. Thus, as the BM occurrence rate in the HAR after HA-WBRT might differ from our results. Second, we did not assess the effect of targeted therapy for BM occurrence in the HAR. Increasing evidence now suggests that tyrosine kinase inhibitors (TKIs) improves progression-free survival in patients with metastatic NSCLC harboring EGFR mutations or ALK rearrangement (7, 64). Moreover, next-generation TKIs show a superior activity in treating BMs (65, 66). Third, the number of patients with BM in HAR is relatively small (n=18). To draw a solid conclusion, further validation in a prospective study with a larger cohort is warranted.

## Conclusion

In conclusion, our study demonstrated that the incidence of BM in the HAR was significantly higher in patients with extra-cranial metastases than in patients without extra-cranial metastases. Age, time interval to BM development, sex, and EGFR mutation status may also affect BM rates in the hippocampal avoidance region. Extending the safety zone from 5 mm to 10 mm in HA-WBRT has little effect on BM incidence in the HAR in patients without extra-cranial metastasis. This study supports the adoption of personalized radiation planning for patients with NSCLC and BMs. These results will allow clinicians to maximize the effectiveness of radiation therapy while minimizing cognitive decline.

## Data Availability Statement

The original contributions presented in the study are included in the article/**Supplementary Material**, further inquiries can be directed to the corresponding author.

## Ethics Statement

The studies involving human participants were reviewed and approved by Gangnam severance hospital institutional review board. Written informed consent for participation was not required for this study in accordance with the national legislation and the institutional requirements.

## Author Contributions

All authors have significantly contributed to the manuscript. Study conception and design, SA and J-ML. Material preparation and data collection, JK and YC. Data analysis, HK and GP. Result interpretation, BJ, MP, and SS. Writing and revision of the manuscript, all authors.

## Funding

This work was supported by Institute of Information & communications Technology Planning & Evaluation (IITP) grant funded by the Korea government (MSIT) [No.2020-0-01373, Artificial Intelligence Graduate School Program(Hanyang University)] to JML and by a National Research Foundation of Korea (NRF) grant funded by the Korea government (MSIT) (No. 2020R1F1A1056512) and grant from the Central Medical Service (CMS) Research Fund to SA.

## Conflict of Interest

The authors declare that the research was conducted in the absence of any commercial or financial relationships that could be construed as a potential conflict of interest.

## Publisher’s Note

All claims expressed in this article are solely those of the authors and do not necessarily represent those of their affiliated organizations, or those of the publisher, the editors and the reviewers. Any product that may be evaluated in this article, or claim that may be made by its manufacturer, is not guaranteed or endorsed by the publisher.
