# Supplementary material for: Hippocampal Metastasis Rate Based on Non-Small Lung Cancer TNM Stage and Molecular Markers
Source: Front Oncol. 2022 May 10;12:781818. doi: 10.3389/fonc.2022.781818 (PMC9127383; doi:10.3389/fonc.2022.781818)
Supplement: Supplementary file 1 [file Table_1.docx]

Supplementary Material

# Supplementary Table

Supplementary Table 1. Multiple logistic regression with backward elimination for BM occurrence in the hippocampus plus 5-mm margin region

|  | OR (95% CI) | p-value |
| --- | --- | --- |
| Smoker | 0.27 (0.06-0.91) | 0.05 |
| Extra-cranial metastasis  at BM occurrence | 8.75 (1.64-162.33) | 0.04* |

Supplementary Table 2. Proportion of hippocampal BM with extended margin in patients without extracranial metastasis

| Hippocampus (N=36) | | | | | |
| --- | --- | --- | --- | --- | --- |
| Region | Estimated proportion (SE) (%) | P-value | Post-hoc analysis | | |
|  |  |  | Region | Diff (SE) | p-value |
| 5-mm margin | 2.78 (2.75) | >0.99 | 5 mm vs. 7.5 mm | 0.00 (0.03) | >0.99 |
| 7.5-mm margin | 2.78 (2.75) |  | 5 mm vs. 10 mm | 0.00 (0.03) | >0.99 |
| 10-mm margin | 2.78 (2.75) |  | 7.5 mm vs. 10 mm | 0.00 (0.03) | >0.99 |

BM: brain metastases

Supplementary Table 3. Basic characteristics of patients who underwent MRI after receiving HA-WBRT

| Patient number | Sex | Age | Histology | TNM | Extra-cranial metastasis | EGFR mutation | ALK Positivity | ROS1 Positivity | Months between RT and MRI | BM in HAR |
| --- | --- | --- | --- | --- | --- | --- | --- | --- | --- | --- |
| 1 | Male | 79 | Large cell carcinoma | 2 | N | Y | N | N | 21 | N |
| 2 | Female | 71 | Adenocarcinoma | 4 | Y | Y | N | N | 14 | N |
| 3 | Male | 81 | Adenocarcinoma | 4 | Y | N | N | N | 2 | N |
| 4 | Female | 61 | Adenocarcinoma | 4 | Y | N | Y | N | 2 | N |
| 5 | Male | 67 | Squamous cell carcinoma | 1 | N | N | N | N/A | 2 | N |
| 6 | Male | 70 | Adenocarcinoma | 3 | N | N | N | N | 26 | N |
| 7 | Female | 57 | Adenocarcinoma | 4 | Y | Y | N | N | 21 | N |
| 8 | Female | 56 | Adenocarcinoma | 3 | N | N | N/A | N/A | 47 | N |
| 9 | Male | 60 | Adenocarcinoma | 4 | Y | N | N | N | 4 | N |
| 10 | Male | 70 | Adenocarcinoma | 4 | Y | Y | N | N/A | 5 | Y |
| 11 | Male | 54 | Adenocarcinoma | 4 | Y | Y | N | N | 3 | N |
| 12 | Male | 74 | Adenocarcinoma | 2 | Y | Y | N/A | N/A | 7 | N |
| 13 | Female | 51 | Adenocarcinoma | 3 | N | N | Y | N | 35 | N |
| 14 | Female | 66 | Adenocarcinoma | 4 | Y | Y | N/A | N/A | 19 | N |
| 15 | Female | 73 | Adenocarcinoma | 4 | Y | Y | N | N/A | 26 | N |
| 16 | Male | 61 | Adenocarcinoma | 3 | Y | Y | N/A | N/A | 6 | Y |
| 17 | Male | 62 | Adenocarcinoma | 3 | N | Y | N/A | N/A | 4 | N |
| 18 | Female | 76 | Adenocarcinoma | 4 | Y | Y | N/A | N/A | 1 | N |
| 19 | Female | 70 | Adenocarcinoma | 3 | N | N | N | N | 2 | N |
| 20 | Male | 62 | Adenocarcinoma | 4 | Y | Y | N | N | 13 | N |
| 21 | Male | 81 | Adenocarcinoma | 4 | Y | N | Y | N | 12 | N |
| 22 | Male | 74 | Adenocarcinoma | 2 | N | N | N | N | 6 | N |
| 23 | Male | 59 | Adenocarcinoma | 4 | Y | Y | N | N | 17 | N |
| 24 | Male | 73 | Adenocarcinoma | 4 | Y | N/A | N/A | N/A | 4 | N |
| 25 | Male | 61 | Large cell carcinoma | 4 | Y | N/A | N/A | N/A | 3 | N |
| 26 | Female | 56 | Adenocarcinoma | 4 | Y | Y | Y | N | 2 | N |

HA-WBRT, hippocampal-avoidance whole-brain radiation therapy; EGFR, epidermal growth factor receptor; ALK, anaplastic lymphoma kinase; MRI, magnetic resonance imaging; BM, brain metastasis; HAR, hippocampal-avoidance region
